# Supplementary material for: Myeloid-derived grancalcin instigates obesity-induced insulin resistance and metabolic inflammation in male mice
Source: Nat Commun. 2024 Jan 2;15:97. doi: 10.1038/s41467-023-43787-x (PMC10762069; doi:10.1038/s41467-023-43787-x)
Supplement: Supplementary file 1 — Supplementary Information [file 41467_2023_43787_MOESM1_ESM.pdf]

Supplementary Materials for

Myeloid-derived grancalcin instigates obesity-induced insulin resistance and metabolic inflammation in male mice

Tian Su[1,#], Yue He[1,#], Yan Huang[1], Mingsheng Ye[1], Qi Guo[1], Ye Xiao[1], Guangping Cai, Linyun Chen[1], Changjun Li, Haiyan Zhou[1,*], Xianghang Luo[1,2,3,5,*]

[1] Endocrinology Research Center, Xiangya Hospital of Central South University, Changsha, Hunan, 410008, China.

[2] National Clinical Research Center for Geriatric Disorders, Xiangya Hospital, Changsha, Hunan 410008, China.

[3] Key Laboratory of Organ Injury, Aging and Regenerative Medicine of Hunan Province, Hunan, 410008, China.

[#]These authors contributed equally

* Corresponding authors

Correspondence and request for reprints should be addressed to:

Prof. Xianghang Luo,

E-mail: xianghangluo@csu.edu.cn;

Dr. Haiyan Zhou,

E-mail: hyzhou02@csu.edu.cn

The PDF file includes:

Supplementary Figures 1 to 8

Supplementary Tables 1 to 2

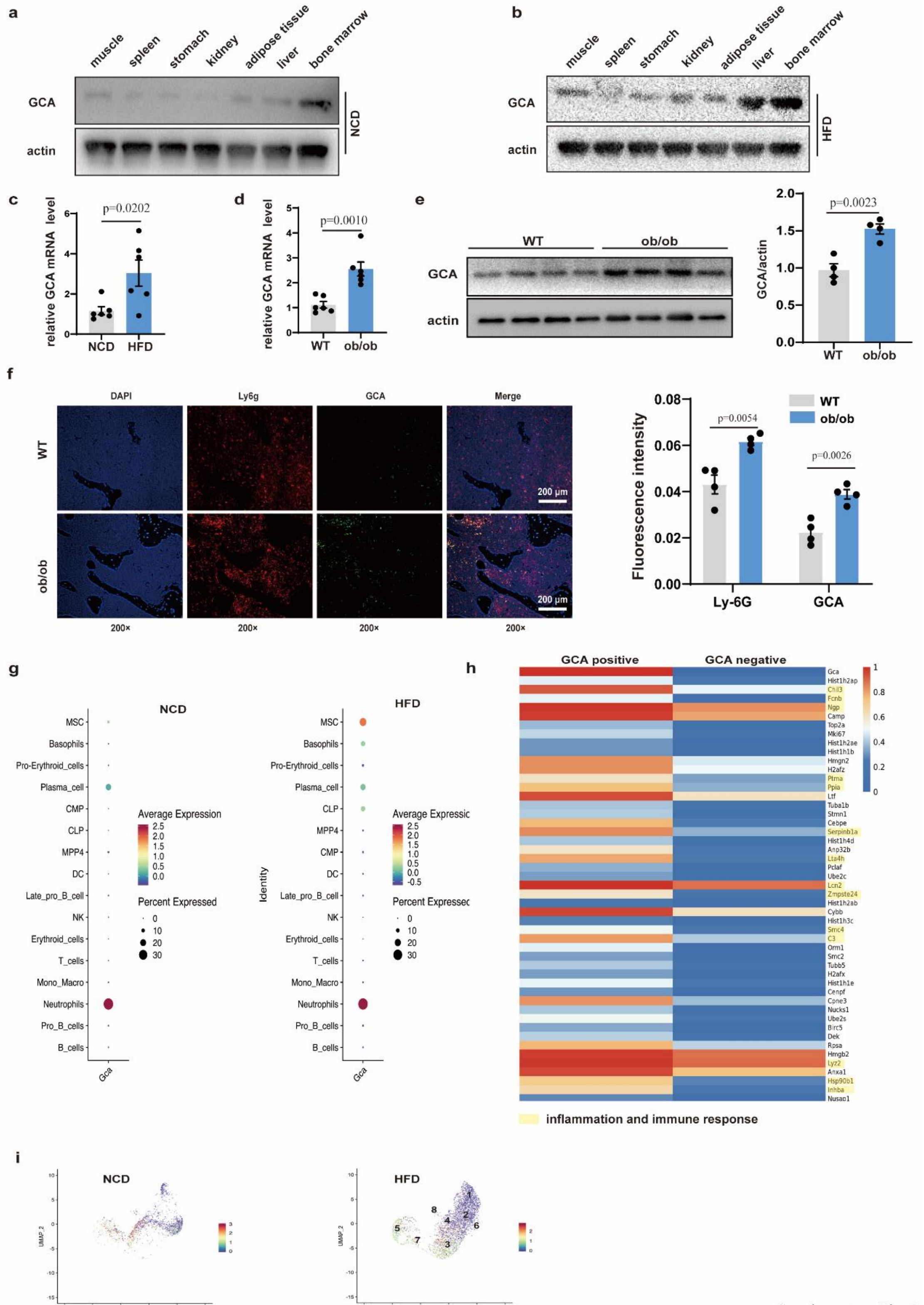

Supplementary Fig. 1

Supplementary Figure 1 Obesity induces a dynamic increase of GCA+ immune cells in the bone marrow.

(a) GCA protein expression levels in multiple tissues of 18 weeks old WT mice fed with NCD (n = 3).

(**b**) GCA protein expression levels in multiple tissues of WT mice fed with HFD for 12 weeks (n = 3).

(c-d) *Gca* mRNA expression levels in the bone marrow of HFD induced mice and genetically obese (ob/ob) mice (n = 6).

(e) Western Blot of GCA protein level in the bone marrow of genetically obese (ob/ob) mice(n=4) (left, representative pictures of Western blot; right, quantitative measurements of GCA proteins).

(f) Immunofluorescent staining of GCA (green) and Ly6g(red) in the bone section of ob/ob mice(n=4) (top, representative pictures of Immunofluorescent staining; bottom, quantitative measurements of Ly6g and GCA proteins). Scale bar as indicated in the picture.

(g) Dotplot shows gene expression of *Gca* genes in cell populations of NCD mice and HFD mice.

(h) The heatmap of 50 most upregulated genes in GCA-positive vs GCA-negative neutrophils.

(i) T-distributed stochastic neighbor embedding (tSNE) plot shows different clustering of bone marrow neutrophils from NCD mice and HFD mice based on gene expression.

Data are presented as means ± SEM. n indicates the number of biologically independent samples examined. Statistical analysis was assessed by two-sided Student's t test and significant differences were indicated with p values. Source data are provided as a Source Data File.

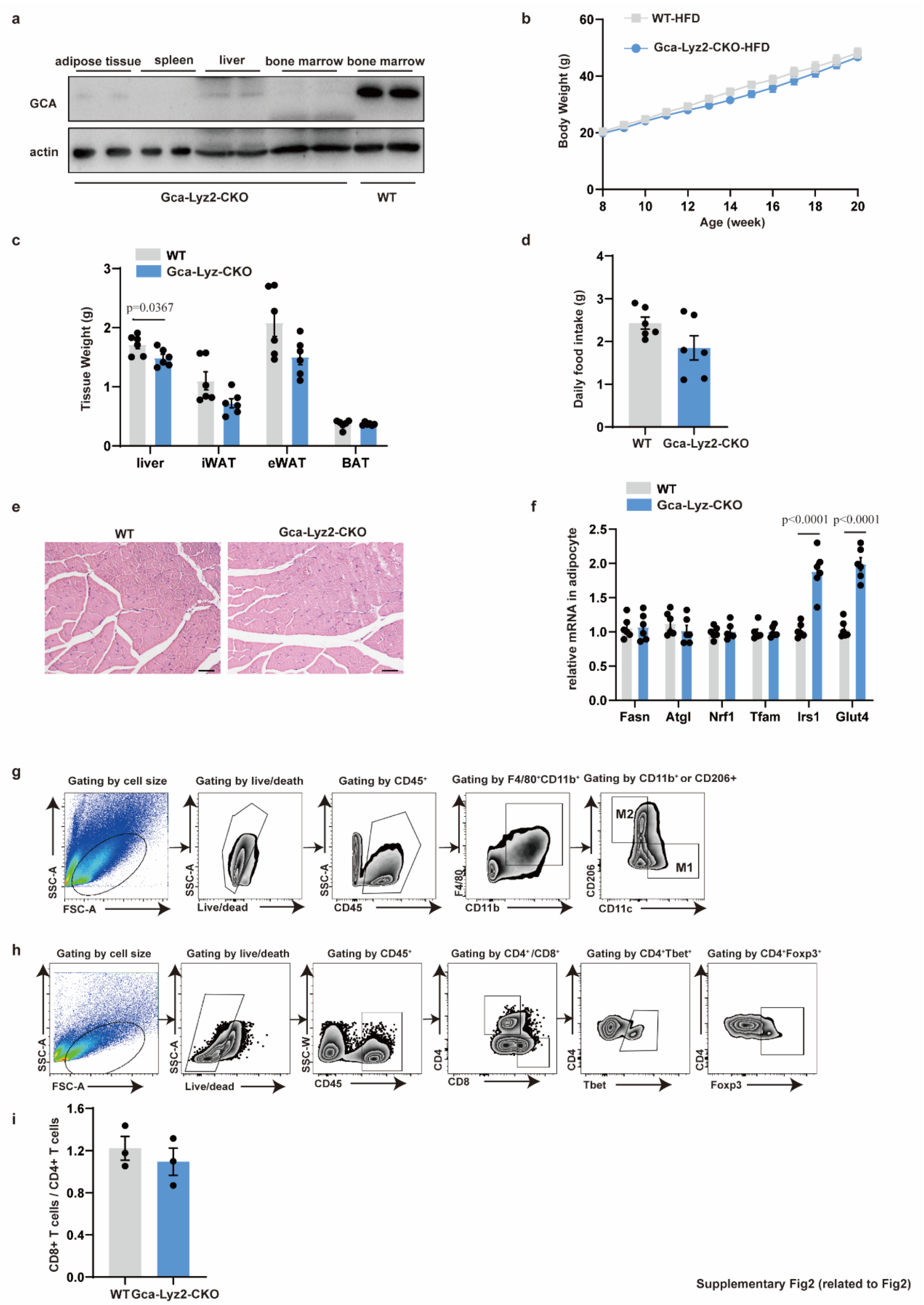

Supplementary Figure 2 GCA deficiency in myeloid lineage meliorates adipose tissue inflammation and glucose metabolism.

(a) GCA expression in bone marrow, eWAT, liver and slpeen of WT and Gca-Lyz2-CKO mice as assessed by western blotting.

(**b**) Body weight of male WT and Gca-Lyz2-CKO mice fed with HFD for 12 weeks (n =6).

(**c**) Weight of liver, eWAT, iWAT and BAT in male WT and Gca-Lyz2-CKO mice fed with HFD for 12 weeks (n =6).

(**d**) The daily food intake of male WT mice and Gca-Lyz2-CKO mice fed with HFD for 12 weeks (n =6).

(**e**) HE staining of muscle from male WT mice and Gca-Lyz2-CKO mice fed with HFD for 12 weeks (n=3).

(f) Relative mRNA levels of lipid synthesis (Fasn), lipolysis (Atgl), mitochondrial biogenesis (Nrf1 and Tfam) and insulin action (Irs1 and Glut4) genes in adipocytes from male WT and Gca-Lyz2-CKO mice fed with HFD for 12 weeks (n =6).

(**g**) The gating strategies for the macrophage flow cytometry as shown in Figure 2o.

(**h**) The gating strategies for the T cells flow cytometry as shown in Figure 2q.

(**i**) Flow cytometry analysis of $CD4^+$ T cells and $CD8^+$ T cell in eWAT from male WT and Gca-Lyz2-CKO mice fed with HFD for 12 weeks (n =3).

Data are presented as means ± SEM. n indicates the number of biologically independent samples examined. Statistical analysis was assessed by two-sided Student's t test (c, d, f and i) or two-way ANOVA followed with Sidak's multiple comparisons test (b) and significant differences were indicated with p values. Source data are provided as a Source Data File.

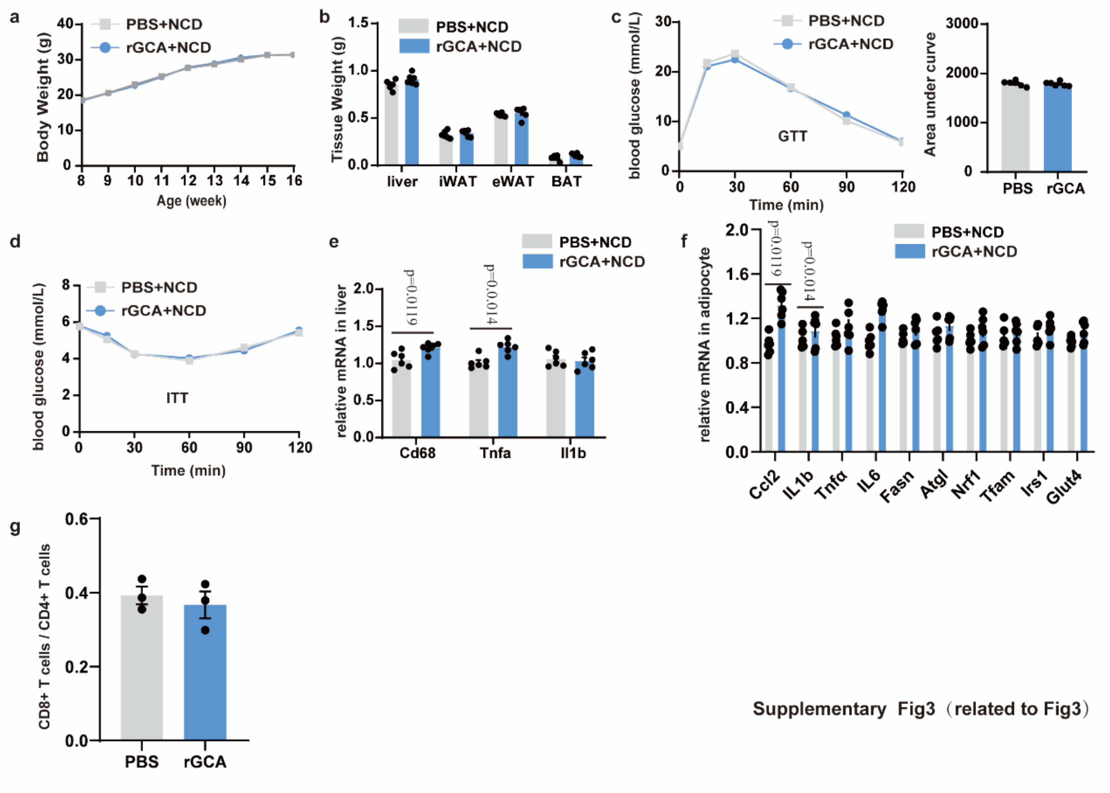

Supplementary Figure 3 GCA exacerbates adipose tissue inflammation in NCD-fed mice.

(a) Body weight gain of NCD-fed animals treated with PBS or rGCA (n=6).

(b) Weight of Liver, eWAT, iWAT and BAT in NCD-fed animals treated with PBS or rGCA (n=6).

(**c-d**) GTT and ITT of NCD-fed mice treated with PBS or rGCA (n = 6).

(e) QPCR analysis of inflammatory cytokine gene expression levels in liver from NCD-fed animals treated with PBS or rGCA (n=6).

(f) Relative mRNA levels of inflammatory cytokine gene, lipid synthesis (Fasn), lipolysis (Atgl), mitochondrial biogenesis (Nrf1 and Tfam) and insulin action (Irs1 and Glut4) genes in adipocytes from NCD-fed animals treated with PBS or rGCA (n=6).

(g) Flow cytometry analysis of $CD4^+$ T cells and $CD8^+$ T cell in eWAT from NCD-fed animals treated with PBS or rGCA (n=3).

Data are presented as means ± SEM. n indicates the number of biologically independent samples examined. Statistical analysis was assessed by two-sided Student's t test (**b**, **c**(right), and **e-g**) or two-way ANOVA followed with Sidak's multiple comparisons test (**a, c**(left) and **d**) and significant differences were indicated with p values. Source data are provided as a Source Data File.

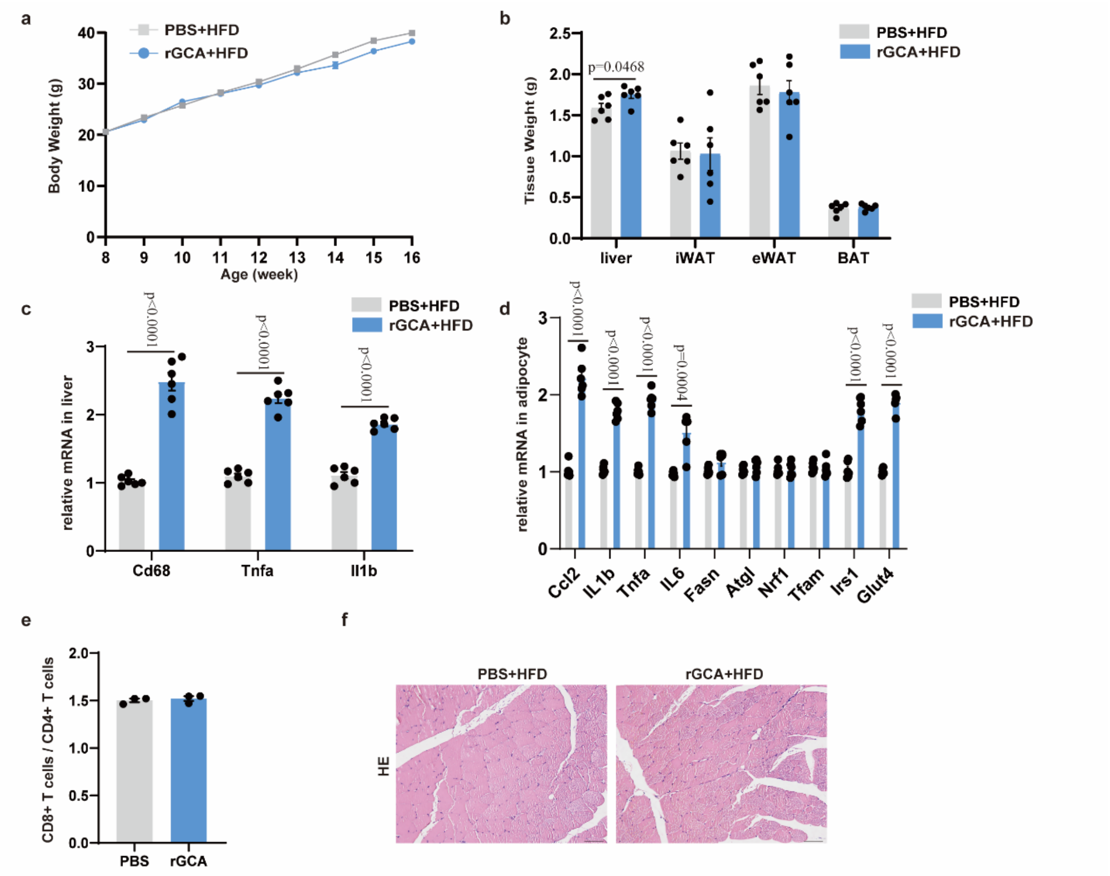

Supplementary Figure 4 GCA exacerbates adipose tissue inflammation, insulin resistance and glucose intolerance in HFD-fed mice.

(a) Body weight gain of HFD-fed animals treated with PBS or rGCA (n=6).

(b) Weight of Liver, eWAT, iWAT and BAT in HFD-fed animals treated with PBS or rGCA (n=6).

(c) QPCR analysis of inflammatory cytokine gene expression levels in liver from HFD-fed animals treated with PBS or rGCA (n=6).

(d) Relative mRNA levels of inflammatory cytokine gene, lipid synthesis (Fasn), lipolysis (Atgl), mitochondrial biogenesis (Nrf1 and Tfam) and insulin action (Irs1 and Glut4) genes in adipocytes from HFD-fed animals treated with PBS or rGCA (n=6).

(e) Flow cytometry analysis of $CD4^{+}$ T cells and $CD8^{+}$ T cell in eWAT from HFD-fed animals treated with PBS or rGCA (n=3).

(f) HE staining of muscle from HFD-fed animals treated with PBS or rGCA (n=3).

Data are presented as means ± SEM. n indicates the number of biologically independent samples examined. Statistical analysis was assessed by two-sided Student's t test (**b-e**) or two-way ANOVA

followed with Sidak's multiple comparisons test (**a**) and significant differences were indicated with p values. Source data are provided as a Source Data File.

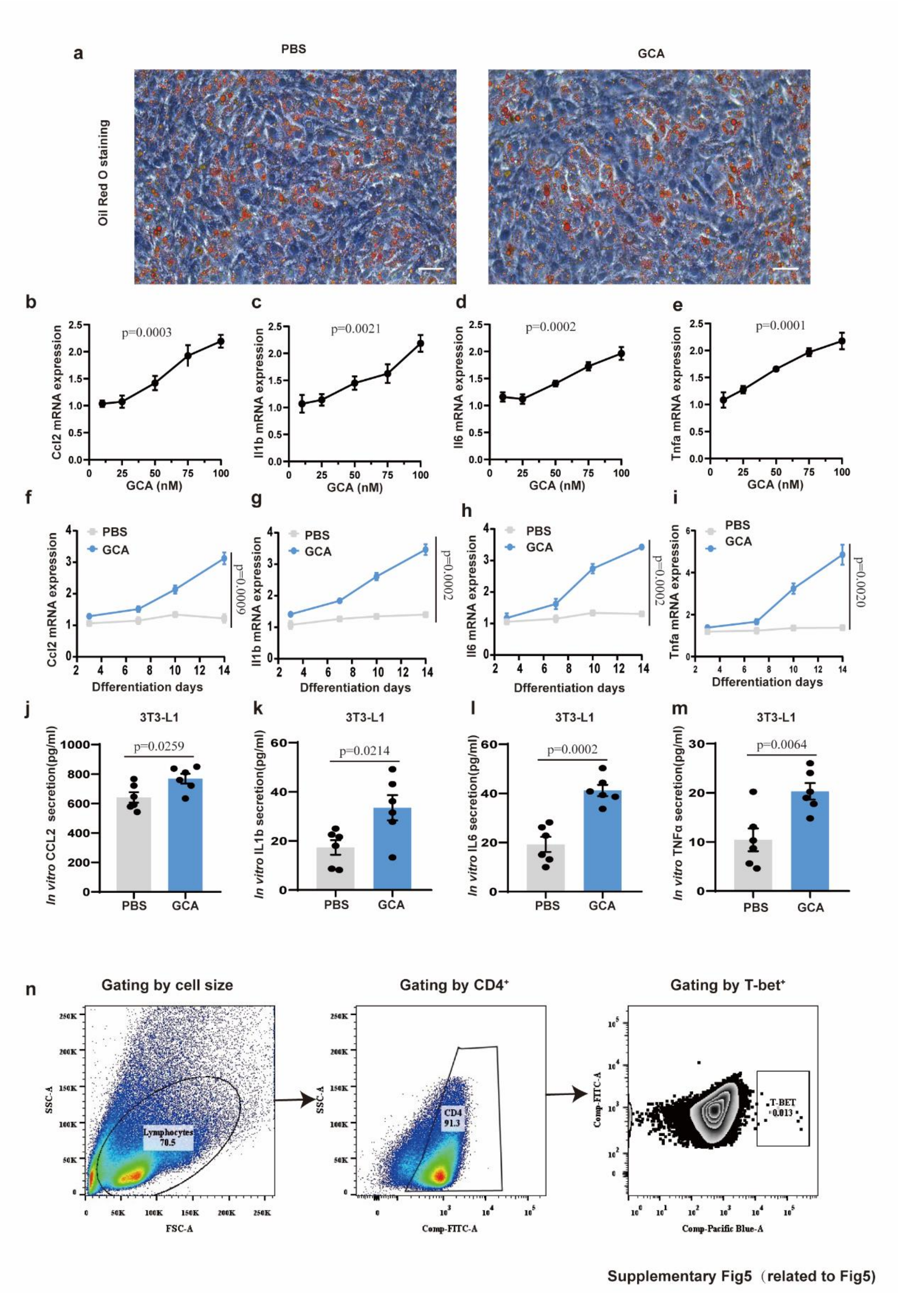

Supplementary Figure 5 GCA magnified inflammation in adipocytes in vitro.

(**a**) Oil Red O staining of 3T3-L1 adipocytes at day 14 of adipogenic induction differentiation process administrated with 100 nM GCA or PBS. Scale bar, 200 µm.

(**b-e**) QPCR analysis of *Ccl2* (**b**), *Il1b* (**c**), *Il6* (**d**) and *Tnfa* (**e**) in differentiated 3T3-L1 adipocytes

administrated with GCA concentrations ranging from 10 to 100 nM.

(**f–i**) QPCR analysis of CCL2 **(f)**, IL1b **(g)**, IL6 **(h)** and TNFα **(i)** expression in 3T3-L1 adipocytes administrated with 100 nM GCA or PBS analyzed at four time points during adipogenic induction differentiation process.

(**j–m)** The protein secretion of CCL2 (**j**), IL1b (**k**), IL6 (**l**) and TNFα(**m**) at day 14 of adipogenic induction differentiation process in 3T3-L1 administrated with 100 nM GCA or PBS.

(**n**) The gating strategies of flow cytometry analyses for T cells after coculture assay.

Data are presented as means ± SEM. n indicates the number of biologically independent samples examined. Statistical analysis was assessed by two-sided Student's t test (**j-m**), one-way ANOVA with Tukey's multiple-comparison test (b-e) or two-way ANOVA followed with Sidak's multiple comparisons test (f-i) and significant differences were indicated with p values. Source data are provided as a Source Data File.

a

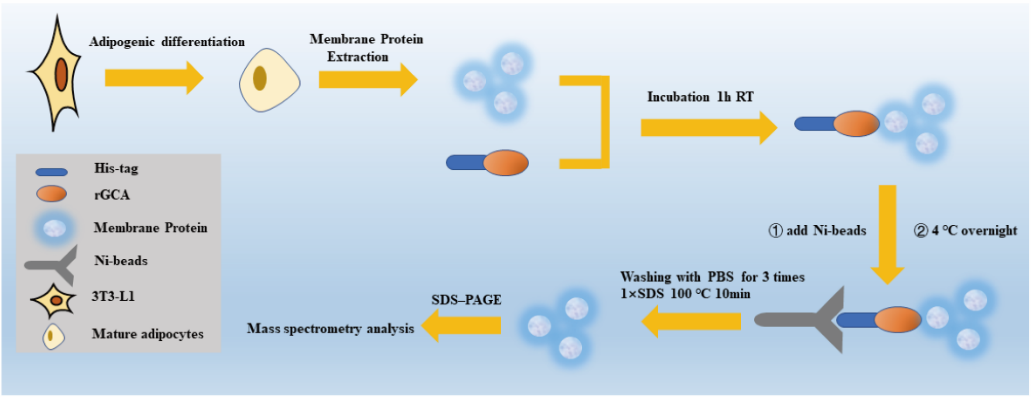

b

Accession	Protein names	Gene names	MW [kDa]	Protein score	Sequence coverage (%)	# Unique Peptides	# Peptides	# PSMs
Q8VDD5	Myosin-9	Myh9	226.23	370.60	6.79	9	10	11
Q7TPR4	Alpha-actinin-1	Actn1	103.00	332.41	10.20	8	8	8
O35129	Prohibitin-2	Phb2	33.28	324.63	27.09	7	7	7
P68033	Actin, alpha cardiac muscle 1	Actc1	41.99	317.50	24.93	1	8	13
P35564	Calnexin	Canx	67.24	315.55	20.30	10	10	12

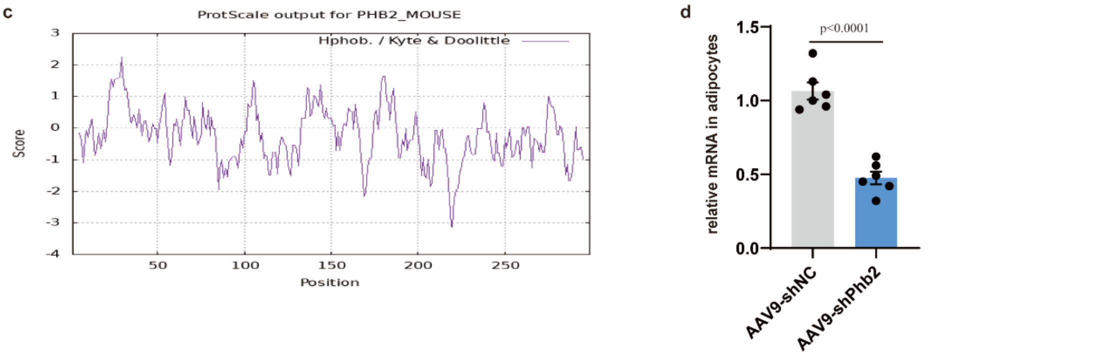

Supplementary Fig6 (related to Fig6)

Supplementary Figure 6 PHB2 is a functional receptor of GCA in adipocyte.

(**a**) Schematic of His-tagged GCA pull-down assays. This diagram was created with MedPeer.com.

(**b**) List of candidates with top 5 scores in LC-MS/MS analysis of 3T3-L1 with incubation of His-labeled GCA.

(c) Transmembrane helix prediction for PHB2 predicted by ProtScale (https://web.expasy.org/protscale/).

(d) qPCR analysis of Phb2 mRNA in AAV9-shNC mice and AAV9-shPhb2 mice one month after virus injection.

Data are presented as means ± SEM. n indicates the number of biologically independent samples examined. Statistical analysis was assessed by two-sided Student's t test and significant differences were indicated with p values. Source data are provided as a Source Data File.

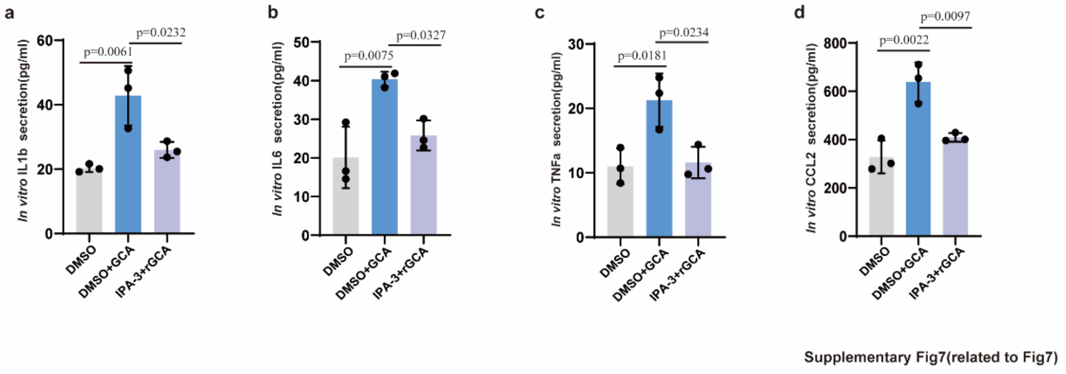

Supplementary Figure 7 GCA promotes phosphorylation of PAK1-NF-κB downstream of PHB2 signaling.

(a-d) ELISA of IL1b **(a)**, IL6 **(b)**, TNFα **(c)** and CCL2 **(d)** in differentiated 3T3-L1 adipocytes with addition of IPA-3 or DMSO and with or without GCA treatment(n=3).

Data are presented as means ± SEM. n indicates the number of biologically independent samples examined. Statistical significance was assessed by one-way ANOVA with Tukey's multiple-comparison test and significant differences were indicated with p values. Source data are provided as a Source Data File.

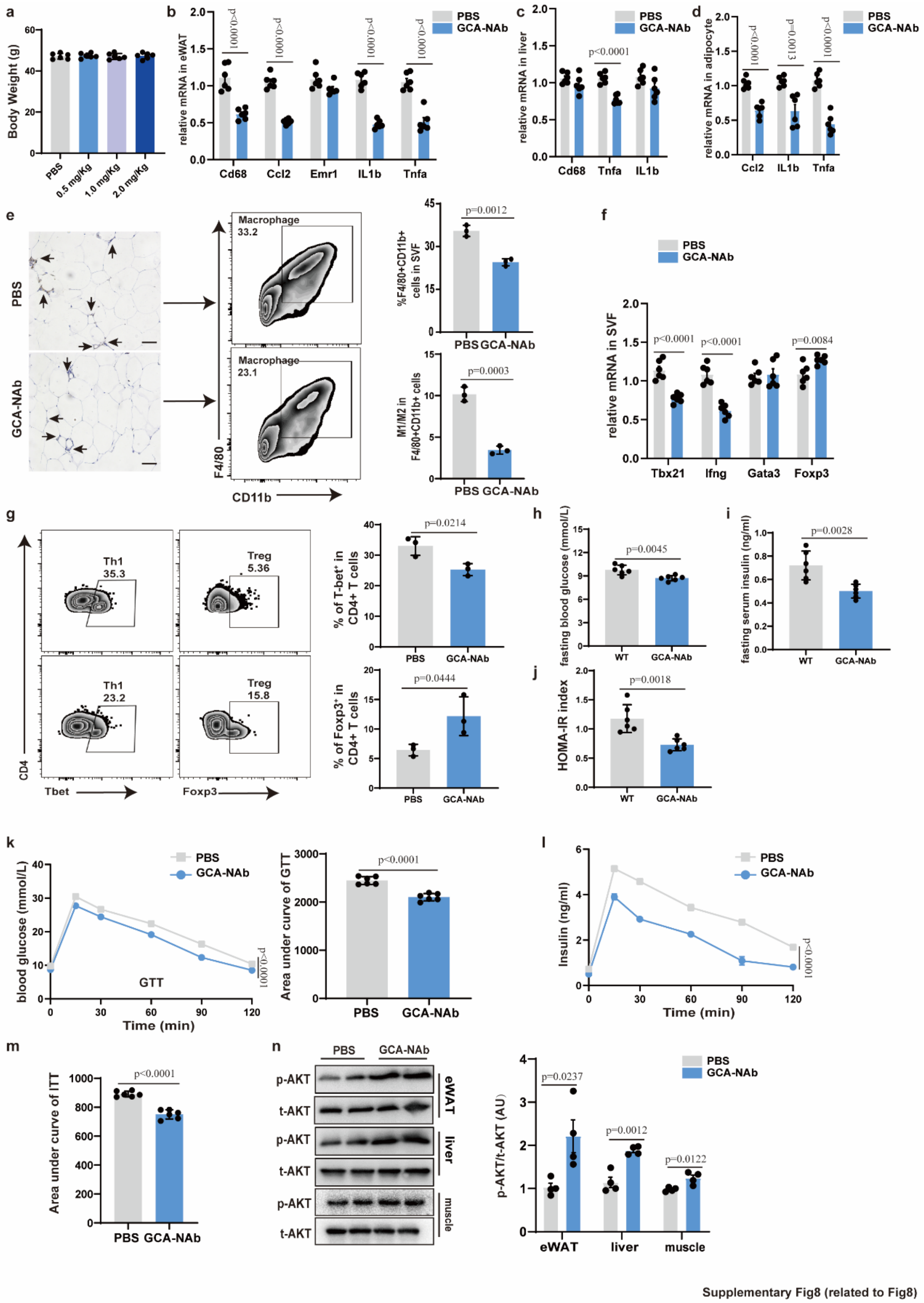

Supplementary Figure 8 GCA-neutralizing antibody improves adipose tissue inflammation and insulin sensitivity in DIO mice.

(a) Body weight of DIO mice treated with PBS or different concentrations of GCA-Nab for 2

months (n=6).

(**b-d**) Inflammatory gene expression in eWAT(**a**), liver(**b**) and adipocyte(**c**) from ob/ob mice treated with PBS or GCA-NAb for 2 months (n=6).

(**e**) F4/80 immunohistochemistry and flow analyses of macrophage in eWAT from ob/ob mice treated with PBS or GCA-NAb for 2 months (n=3). Scale bar, 250μm.

(**f**) Gene expression in SVF from ob/ob mice treated with PBS or GCA-NAb for 2 months (n=6).

(**g**) Flow cytometry analysis of $CD4^+Tbet^+$ Th1 cells and $CD4^+Foxp3^+$ Treg cells in eWAT from ob/ob mice treated with PBS or GCA-NAb for 2 months (n=3).

(**h-j**) Fasting blood glucose, fasting insulin and HOMA-IR index of ob/ob mice treated with PBS or GCA-NAb for 2 months (n = 6).

(**k-l**) Time courses of blood glucose and serum insulin concentrations in ob/ob mice treated with PBS or GCA-NAb for 2 months (n = 6).

(m) ITT of ob/ob mice treated with PBS or GCA-NAb for 2 months (n = 6).

(**n**) Western Blot of insulin-stimulated AKT phosphorylation in eWAT, liver and muscle, and quantitation of pAKT/tAKT from ob/ob mice treated with PBS or GCA-NAb for 2 months (n=4).

Data are presented as means ± SEM. n indicates the number of biologically independent samples examined. Statistical analysis was assessed by one-way ANOVA with Tukey's multiple-comparison test (**a**), two-sided Student's t test (**b-j**, **k**(right), **m** and **n**) or two-way ANOVA followed with Sidak's multiple comparisons test (**k（left）**, **l**) and significant differences were indicated with p values. Source data are provided as a Source Data File.

Supplementary Table 1 Characteristics of the Study Population

Trait	individuals without obesity (n=12)	individuals with obesity (n=38)
Age (years)	25.75±2.20	29.50±8.02
Male/female (n)	6/6	14/24
BMI (kg/m^2)	20.19±1.69	29.04±0.93
HOMA-IR	1.04±0.07	7.12±6.23
Fasting blood glucose (mmol/l)	4.49±0.17	5.77±0.88
2h blood glucose (mmol/l)	5.49±0.28	8.44±2.89
Fasting insulin (μU/ml)	5.20±0.32	26.72±21.30
2h insulin(μU/ml)	23.35±3.66	174.44±139.83
Cholesterol (mmol/l)	4.54±0.53	5.91±0.77
Triglycerides (mmol/l)	1.48±0.11	2.23±1.11

Data are presented as mean±SEM.

Supplementary Table 2 Nucleotide sequences of primers used for quantitative RT-PCR detection in mouse

primer	sequence (5' to 3')	
	Forward	Reverse
Cd68	TGTCTGATCTTGCTAGGACCG	GAGAGTAACGGCCTTTTTGTGA
Ccl2	TTAAAAACCTGGATCGGAACCAA	GCATTAGCTTCAGATTTACGGGT
IL1b	CCCTGCAGCTGGAGAGTGTGGA	CTGAGCGACCTGTCTTGGCCG
Emr1	TGACTCACCTTGTGGTCCTAA	CTTCCCAGAATCCAGTCTTTCC
IL6	GCCCATCCTCTGTGACTCAT	CAGAATTGCCATTGCACAAC
Tnfα	AAGCCTGTAGCCCACGTCGTA	GGCACCACTAGTTGGTTGTCTTTG
Gca	GGGGCGTTTGGAAACTTCAG	AGGGGAGTAGCTGTCAGAATAAC
Ciita	GCTGGGACGAAGACACCAA	CACCTCCACGGATGAAAACC
H2-EB1	ACAGCCCAATGTCGTCATCTC	CCAGAGTGTTGTGGTGGTTGA
Cd74	CGCGACCTCATCTCTAACCAT	ACAGGTTTGGCAGATTTCGGA
Tbx21	AGCAAGGACGGCGAATGTT	GTGGACATATAAGCGGTTCCC
Ifng	ATGAACGCTACACACTGCATC	CCATCCTTTTGCCAGTTCCTC
Gata3	CCAGGCAAGATGAGAAAGAGTG	ATAGGGCGGATAGGTGGTAATG
Foxp3	ACTCGCATGTTCGCCTACTT	AGGGATTGGAGCACTTGTTG
Fasn	AGAGATCCCGAGACGCTTCT	GCTTGGTCCTTTGAAGTCGAAGA
Atgl	CAAGGGGTGCGCTATGTGGATGG	GAGGCGGTAGAGATTGCGAAGGTT
Nrf1	AGCACGGAGTGACCCAAAC	TGTACGTGGCTACATGGACCT
Tfam	ATTCCGAAGTGTTTTTCCAGCA	TCTGAAAGTTTTGCATCTGGGT
Irs1	GCCAGAGGATCGTCAATAGC	AAGACGTGAGGTCCTGGTTG
Glut4	TTGGAGAGAGAGCGTCCAAT	TTGATGCCTGAGAGCTGTTG
