# Supplementary material for: Myeloid-derived grancalcin instigates obesity-induced insulin resistance and metabolic inflammation in male mice
Source: Nat Commun. 2024 Jan 2;15:97. doi: 10.1038/s41467-023-43787-x (PMC10762069; doi:10.1038/s41467-023-43787-x)
Supplement: Supplementary file 3 — Reporting Summary [file 41467_2023_43787_MOESM3_ESM.pdf]

nature portfolio

Corresponding author(s): Xianghang Luo

Last updated by author(s): Sep 28, 2023

Reporting Summary

Nature Portfolio wishes to improve the reproducibility of the work that we publish. This form provides structure for consistency and transparency in reporting. For further information on Nature Portfolio policies, see our Editorial Policies and the Editorial Policy Checklist.

Statistics

For all statistical analyses, confirm that the following items are present in the figure legend, table legend, main text, or Methods section.

n/a	Confirmed	
☐	☒	The exact sample size (*n*) for each experimental group/condition, given as a discrete number and unit of measurement
☐	☒	A statement on whether measurements were taken from distinct samples or whether the same sample was measured repeatedly
☐	☒	The statistical test(s) used AND whether they are one- or two-sided<br>*Only common tests should be described solely by name; describe more complex techniques in the Methods section.*
☒	☐	A description of all covariates tested
☐	☒	A description of any assumptions or corrections, such as tests of normality and adjustment for multiple comparisons
☐	☒	A full description of the statistical parameters including central tendency (e.g. means) or other basic estimates (e.g. regression coefficient) AND variation (e.g. standard deviation) or associated estimates of uncertainty (e.g. confidence intervals)
☐	☒	For null hypothesis testing, the test statistic (e.g. *F*, *t*, *r*) with confidence intervals, effect sizes, degrees of freedom and *P* value noted<br>*Give P values as exact values whenever suitable.*
☒	☐	For Bayesian analysis, information on the choice of priors and Markov chain Monte Carlo settings
☒	☐	For hierarchical and complex designs, identification of the appropriate level for tests and full reporting of outcomes
☐	☒	Estimates of effect sizes (e.g. Cohen's *d*, Pearson's *r*), indicating how they were calculated

Our web collection on statistics for biologists contains articles on many of the points above.

Software and code

Policy information about availability of computer code

Data collection	BD FACS Diva software v9.0 and BD Canto II/Aria system(flow cytometry), Applied Biosystem 7900HT Real-Time PCR System (qPCR), OLYMPUS CX41-32RFL microscope system and Leica SP8 confocal microscope system(immunofluorescence).Western Blot images were captured by Image Lab version 4.1(Bio-Rad).
Data analysis	FlowJo VlO (flow cytometry),GraphPad Prism 8 .0 (data analysis), ImageJ 1.44 (imaging analysis), featureCounts software, Seurat V3.l.2, R package clusterProfiler (Version 3.5.1)

For manuscripts utilizing custom algorithms or software that are central to the research but not yet described in published literature, software must be made available to editors and reviewers. We strongly encourage code deposition in a community repository (e.g. GitHub). See the Nature Portfolio guidelines for submitting code & software for further information.

Data

Policy information about availability of data

All manuscripts must include a data availability statement. This statement should provide the following information, where applicable:

- Accession codes, unique identifiers, or web links for publicly available datasets
- A description of any restrictions on data availability
- For clinical datasets or third party data, please ensure that the statement adheres to our policy

The authors declare that the main data supporting the findings of this study are available within the article and its Supplementary information. The source data are

provided with the paper as a Source Data file.

Human research participants

Policy information about studies involving human research participants and Sex and Gender in Research.

Reporting on sex and gender	Study participants included 38 obese patients（24 females and 14 males） and 12 nonobese controls(6 females and 6 males).
Population characteristics	Human peripheral blood were colloceted from people defined healthy status based on no prior history of thyroid diseases, autoimmune disease, taking medications, malignant tumors, other endocrinological diseases, such as Cushing syndrome, vital organ failure, such as heart, liver or renal. and divided into two categories based on BMI>28kg/m2; nonobese: BMI<28kg/m2.
Recruitment	The participants were randomly recruited at Xiangya Hospital of Central South University. There is no potential self-selection biasor other biases.All participants recruited in the study signed informed consent. The obese patients were recruited from out-patient department who were diagnosed as obesity. The nonobese controls were recruited from healthy people.
Ethics oversight	The Ethics Committee of Xiangya Hospital at Central South University. And the protocols followed were compliant with the ethical principles of the Helsinki Declaration. Written informed consents were obtained from all participants.

Note that full information on the approval of the study protocol must also be provided in the manuscript.

Field-specific reporting

Please select the one below that is the best fit for your research. If you are not sure, read the appropriate sections before making your selection.

- [x] Life sciences
- [ ] Behavioural & social sciences
- [ ] Ecological, evolutionary & environmental sciences

For a reference copy of the document with all sections, see nature.com/documents/nr-reporting-summary-flat.pdf

Life sciences study design

All studies must disclose on these points even when the disclosure is negative.

Sample size	Sample size was not predetermined but we performed experiments with group sizes based on existing published literature of similar experiments.For animal experiments, n≥4 was chosen based on the previous publications in the field (Meilian Liu et al, 2014,Cell metabolism).For experiments other than those involving animals,n≥3 was chosen based on the previous publications in thefield(Tuo Denget al.,2013,Cell metabolism) and also because this size is necessary to calculate statistical significances.
Data exclusions	No data were excluded from analysis.
Replication	All experimental findings were reliably reproduced for three times and all replication attempts were successful.
Randomization	All the samples, organisms and participants were randomly allocated into different experimental groups.
Blinding	Investigators were not blinded to treatments, because the investigators who performed the experiments was the person making the analysis and plans. However, no subjective assessments were made.

Reporting for specific materials, systems and methods

We require information from authors about some types of materials, experimental systems and methods used in many studies. Here, indicate whether each material, system or method listed is relevant to your study. If you are not sure if a list item applies to your research, read the appropriate section before selecting a response.

Materials & experimental systems

n/a	Involved in the study	
[ ]	[x]	Antibodies
[ ]	[x]	Eukaryotic cell lines
[x]	[ ]	Palaeontology and archaeology
[ ]	[x]	Animals and other organisms
[x]	[ ]	Clinical data
[x]	[ ]	Dual use research of concern

Methods

n/a	Involved in the study	
[x]	[ ]	ChIP-seq
[ ]	[x]	Flow cytometry
[x]	[ ]	MRI-based neuroimaging

Antibodies

Antibodies used	For immunoprecipitation :HA (H9658, Sigma,1:100); MYC(2276S, Cell Signaling Technology,1:250);<br>For immunoblot :GCA (PA5-77127, Invitrogen,1:1000); p-AKT(CST9271s, Cell Signaling Technology,1:1000); AKT(CST9272s, Cell Signaling Technology,1:1000); PAK1(2602T, Cell Signaling Technology,1:1000); Phospho-PAK1(2601T, Cell Signaling Technology,1:1000); Phospho-NF-κB p65(CST3033S, Cell Signaling Technology,1:1000); NF-κB p65(CST8242S, Cell Signaling Technology,1:1000); PHB2,(sc-133094, Santa Cruz,1:1000).<br>For immunofluorescence Staining:GCA(PA5-77127, Invitrogen,1:200);PHB2(sc-133094, Santa Cruz,1:200); P65(CST8242S, Cell Signaling Technology,1:400); Biotin anti-mouse Ly-6G/Ly-6C (Gr-1) Antibody(Biolegend,108403,1:200);Perilipin-1,Cell Signaling Technology,9349S,1:200) .<br>Immunohistochemistry:F4/80(ab6640, abcam,1:200).<br>For flow cytometry:Brilliant Violet 421™ anti-T-bet Antibody(Biolegend,644832,1:50);FITC anti-mouse CD4 (Biolegend,100509,1:100);APC-Cy7-anti-mouse-CD45(Biolegend,103114,1:100) ;Percp-Cy5.5-anti-mouse-CD3 (Biolegend,100328,1:100); Alexa Fluor® 647 anti-mouse/rat/human FOXP3 Antibody(Biolegend,320014,1:16); APC anti-mouse CD206 (MMR) Antibody(Biolegend,141708,1:40); APC/Cyanine7 anti-mouse CD11c Antibody(Biolegend,117323,1:20); PE anti-mouse F4/80 Antibody(Biolegend,123110,1:20); APC/Cyanine7 anti-mouse CD8a Antibody(Biolegend,100714,1:25); FITC anti-mouse/human CD11b Antibody(Biolegend,101206,1:200).
Validation	For immunoprecipitation :HA (H9658, Sigma,1:200); MYC(2276S, Cell Signaling Technology,1:250);<br>For immunoblot :GCA (PA5-77127, Invitrogen,1:1000); p-AKT(CST9271s, Cell Signaling Technology,1:1000); AKT(CST9272s, Cell Signaling Technology,1:1000); PAK1(2602T, Cell Signaling Technology,1:1000); Phospho-PAK1(2601T, Cell Signaling Technology,1:1000); Phospho-NF-κB p65(CST3033S, Cell Signaling Technology,1:1000); NF-κB p65,(CST8242S, Cell Signaling Technology,1:1000); PHB2,(sc-133094, Santa Cruz,1:1000)were validated by the companies and by users.<br>For immunofluorescence Staining:GCA(PA5-77127, Invitrogen,1:200);PHB2(sc-133094, Santa Cruz,1:200); P65(CST8242S, Cell Signaling Technology,1:400); Biotin anti-mouse Ly-6G/Ly-6C (Gr-1) Antibody(Biolegend,108403,1:200);Perilipin-1,Cell Signaling Technology,9349S,1:200) were validated by the companies and by users.<br>For immunohistochemistry:F4/80(ab6640, abcam,1:200)were validated by the companies and by users.<br>For flow cytometry:Brilliant Violet 421™ anti-T-bet Antibody(Biolegend,644832,1:50);FITC anti-mouse CD4 (Biolegend,100509,1:100);APC-Cy7-anti-mouse-CD45(Biolegend,103114,1:100) ;Percp-Cy5.5-anti-mouse-CD3 (Biolegend,100328,1:100); Alexa Fluor® 647 anti-mouse/rat/human FOXP3 Antibody(Biolegend,320014,1:16); APC anti-mouse CD206 (MMR) Antibody(Biolegend,141708,1:40); APC/Cyanine7 anti-mouse CD11c Antibody(Biolegend,117323,1:20); PE anti-mouse F4/80 Antibody(Biolegend,123110,1:20); APC/Cyanine7 anti-mouse CD8a Antibody(Biolegend,100714,1:25); FITC anti-mouse/human CD11b Antibody(Biolegend,101206,1:200)were validated by the companies and by users.

Eukaryotic cell lines

Policy information about cell lines and Sex and Gender in Research

Cell line source(s)	3T3-L1 preadipocytes were purchased from Procell Life Science & Technology Co. Ltd.(Wuhan,China).
Authentication	3T3-L1 preadipocytes were purchased directly from Procell Life Science&Technology Co.,Ltd and have been authenticated by Procell. The cell line was authenticated by lipid accumulation on adipocyte differentiation, cell morphology by microscopy,and lipogenic gene expression by qPCR analyses.
Mycoplasma contamination	3T3-L1 preadipocytes were tested negative for mycoplasma contamination.
Commonly misidentified lines<br>(See ICLAC register)	No commonly misidentified lines were used.

Animals and other research organisms

Policy information about studies involving animals; ARRIVE guidelines recommended for reporting animal research, and Sex and Gender in Research

Laboratory animals	Mus musculus<br>Wild-type mice, C57BL/6l background,8-week-old male mice were used.Ob/ob ,8-week-old male mice were used.Lyz2-Cre mice , C57BL/6J background, 8 week-old male mice were used to cross with GCA flox/flox mice.Gca-myeloid cell–specific KO mice (gca-/-),C57BL/6l background, 8 week-old male mice were used.Gca flox/flox mice, C57BL/6J background,8 week-old male mice were used.Adipocyte-specific Phb2 knockdown , 8 week-old male mice were used.<br>Mice were bred under specific-pathogen-free conditions at the Laboratory Animal Research Center of Central South University at acontrolled temperature (22-24RC) and humidity (50-50%), with a 12h dark/light cycle (07:00 to 19:00 light on), with standard foodand water provided ad libitum.
Wild animals	This study did not involve wild animals.
Reporting on sex	Only male mice were used in the studies.
Field-collected samples	This study did not include field -collected samples.
Ethics oversight	Care of experimental animals was in accordance with guidelines and approved by the Animal Care and Use Committees of the Laboratory Animal Research Center at Xiangya Medical School of Central South University.

Note that full information on the approval of the study protocol must also be provided in the manuscript.

Flow Cytometry

Plots

Confirm that:

- [x] The axis labels state the marker and fluorochrome used (e.g. CD4-FITC).
- [x] The axis scales are clearly visible. Include numbers along axes only for bottom left plot of group (a 'group' is an analysis of identical markers).
- [x] All plots are contour plots with outliers or pseudocolor plots.
- [x] A numerical value for number of cells or percentage (with statistics) is provided.

Methodology

Sample preparation	eWAT (epidydimal white adipose tissue) was separated from male mice, and washed in PBS with 1% PS. Then putting the chopped tissue in 2 mg/ml collagenase type 2 (C6885, Sigma-Aldrich) in digestion buffer for 30 min at 37 ℃ in a shaker (140 rpm). Adding equal volume of DMEM containing 10% fetal bovine serum (FBS) to stop the digestion of collagenase. The cell suspension was centrifugated at a speed of 1000 rpm for 5 minutes. SVF cells exist in the precipitates. Resuspending the pelleted cells, and filtered through sterile 100 μm mesh filters.
Instrument	BD FCACSCANTOII Flow Cytometry
Software	FlowJo version 10.7.1
Cell population abundance	No post-sorting analysis was done.
Gating strategy	FACS gating was set on FSC and SSC to exclude aggregates. Live cells were further selected by live/death staining. The live cells were further selected according to the different fluorescent channels as depicted in Supplementary Figure2. Representative images were generated with FlowJo version 10.7.1.

- [x] Tick this box to confirm that a figure exemplifying the gating strategy is provided in the Supplementary Information.
